# Risk and Protective Factors for Child Overweight/Obesity Among Low Socio-Economic Populations in Israel: A Cross Sectional Study

**DOI:** 10.3389/fendo.2018.00456

**Published:** 2018-08-21

**Authors:** Varda Soskolne, Michal Cohen-Dar, Samira Obeid, Nitsa Cohen, Mary C. J. Rudolf

**Affiliations:** ^1^Louis and Gabi Weisfeld School of Social Work, Bar-Ilan University, Ramat Gan, Israel; ^2^Northern Region Health Office, Ministry of Health, Nazareth Illit, Israel; ^3^Nursing Department, Max Stern Yezreel Valley College, Emek Yezreel, Israel; ^4^Department of Population Health, Azrieli Faculty of Medicine, Bar-Ilan University, Safed, Israel

**Keywords:** risk factors, protective factors, child obesity, preschool, ethnic differences, social disadvantage, Israel

## Abstract

**Background and Aims:** Scientific evidence regarding protective factors that contribute to healthy weight in childhood is limited and is particularly scarce in lower socio-economic populations in different ethnic groups. This study aimed to assess the prevalence of biological, behavioral and psychosocial factors for child overweight/obesity in Jewish and Arab population groups in Israel, and to compare their associations with child overweight/obesity in the two groups.

**Methods:** Children aged 5–6 years were randomly selected from 20 Mother and Child Health clinics in towns and villages of lowest socio-economic ranking in Northern Israel. Children and mothers were invited for a special “One Stop Shop–Preparation for School” visit which included growth measurements. Questionnaires were distributed to mothers for self-report on biological, SES, psychological and lifestyle factors. Perinatal and early nutritional data were retrieved from clinic records. Multivariate analyses using logistic regression models predicting child overweight/obesity were conducted separately for Jewish (*N* = 371) and Arab (*N* = 575) children.

**Results:** Overweight/obesity (BMI ≥85th centile) rates were higher in Jewish (25%) than Arab (19%) children. In both Jewish and Arab groups, respectively, maternal BMI (OR = 1.10 [95%CI = 1.04, 1.17]; OR = 1.08 [95%CI = 1.04, 1.13]), and child birthweight (OR = 1.33 [95%CI = 1.04, 1.71]; OR = 1.39 [95%CI = 1.11, 1.73]) were significant risk factors for overweight/obesity, and maternal self-efficacy regarding child's lifestyle was significantly protective (OR = 0.49 [95%CI = 0.28, 0.85]; OR = 0.54 [95%CI = 0.34, 0.85]). Additionally, four other maternal psychological and child behaviors were significantly associated with overweight/obesity in the Jewish group and two child lifestyle behavior factors in the Arab group. Moreover, significant interactions indicating moderation effects were found only in the Jewish group: maternal education and maternal age moderated the effect of maternal BMI on child overweight/obesity. No other moderation of risk factors was found.

**Discussion:** In this study of children from low SES families, protective factors contributed to healthy child weight alongside risk factors for overweight/obesity. They differed between the population groups, and fewer variables explained overweight/obesity in Arab children. Although further expansion of these findings is required they point at the relevance of protective factors, maternal self-efficacy in particular, for understanding childhood obesity in specific ethnic contexts and for planning culturally adapted prevention programs in disadvantaged populations.

## Introduction

The increase in levels of overweight and obesity from a young age poses a challenge for public health throughout the world (1). The short-term consequences include greater risk for children's physical health, and social and psychological problems in childhood (2). Children with obesity are more likely to grow up to be adolescents and adults with obesity, with health and economic consequences (3–5). Moreover, it is evident that complications previously occurring only in older age now appear in childhood (6), and that disparities in child obesity between population groups, such as between race/ethnic majority and minorities, and by socio-economic status (SES) are already manifest (4, 7, 8). Within the Israeli context, child overweight and obesity has increased in the past 12 years, and inequalities exist between Jewish and Arab children (9). The current study focused on these two population groups.

Overweight in children is defined as body mass index (BMI) for age and sex centile (≥85–<95%), and obesity (≥95%) (10). The prevalence of overweight (including obesity) for school-age children in OECD countries is 25%, with large differences between countries (11). Particularly alarming is the evidence that overweight and obesity rates according to national growth references are already very high in preschool children; as high as 27% in the US (4), 25% in Sweden (8), 20% in the UK (12), yet only 9% in China (13). Understanding the factors that explain obesity in early childhood is important, particularly the modifiable characteristics, so that efforts to prevent obesity and reduce gaps can begin before school age.

The causes of child obesity are complex and include genetic, biological, psychosocial, behavioral and cultural factors (14). Building upon an ecological approach, a biopsychosocial framework to understanding obesity suggests that biological (nature) and psychosocial and behavioral factors (nurture) are both involved in child weight status (15). They include child characteristics, such as gender and age, birth weight, dietary intake and activity behaviors; parenting styles and family characteristics, such as parental lifestyles, monitoring child activity and diet, parental weight status; and demographic, community and societal characteristics, such as SES, ethnicity, crime rates and neighborhood safety, leisure time, work hours (15). The current study follows this framework by inclusion of a combination of child and maternal biological, socio-economic, psychological and behavioral factors in explaining overweight and obesity among preschoolers aged 5–6 years old. In light of the known differences in child obesity between ethnic populations (4, 16, 17), including in Israel (9), we opted to examine this framework separately for each of the two population groups.

A range of biological, socioeconomic, behavioral and psychosocial factors were selected for our study. There is strong and consistent evidence regarding genetic and biological determinants of obesity (hereafter biological). It shows an increased likelihood of childhood overweight and obesity when one (generally the mother) or both parents are overweight (8, 18). Among the child factors, higher birth weight is a consistent predictor of obesity (4) while the evidence regarding early nutrition during infancy is less consistent. In some studies breastfeeding has been shown to be protective against child obesity whereas others have shown no or little effect (15, 19).

SES, a major social determinant of health (20), has been significantly associated with child overweight and obesity in the majority of studies. Rates of obesity increase with decline in SES measured by diverse measures, such as social class, income and maternal education (21). This association has been shown among 0–8 years old children (7), children aged 4–5 years (22), and even before the age of four (23). Yet, a review of studies from high-income countries showed that the association was evident in less than half of the studies (24).

SES and ethnicity reflect the environment in which children grow, particularly the most proximal environment, that of the family. The health behaviors of the parents, particularly those of the mother, and the health behavior of the child have been identified as major risk factors for child obesity. These mainly include the child's diet (high-fat and sugary foods/drinks (5, 25), child and maternal physical activity and sedentary behavior (26, 27), child behavior problems (25, 28) and maternal smoking during pregnancy and childhood (23). In addition, psychological cognitive factors, particularly maternal self-efficacy regarding children's health behaviors, have been shown to reduce children's excessive food intake and obesity (17, 29).

Several studies have shown that behavioral risk factors for child obesity are more prevalent in lower SES classes (7, 30). Others argue that to fully understand factors that explain the socio-economic disparities in child obesity, research should focus especially on children from low socio-economic backgrounds (31), an approach adopted in the present study. The few studies that have focused only on children from low SES populations show that biological risk factors [maternal BMI and birth weight (32) and, for example, a combination of social risk and behavioral problems (25) or family food behaviors and maternal depression (33)] are associated with child obesity. In a systematic review of the effects of child and parent behaviors on child obesity in children from disadvantaged backgrounds, Russel et al. (31) found that most studies have focused on risk factors such as maternal weight, child physical and sedentary activity, or feeding practices, and that clustering of diet, weight and feeding behaviors by socio-economic indicators precluded identification of independent effects of each of these risk factors. Others have stressed that studies on SES disparities in child obesity rarely investigate parental psychological attributes such as attitudes, beliefs, self-esteem and so on as potential factors in each socio-economic group (7). Thus, examining the socio-economic, diet and behavioral factors separately and extending to include psychological factors should be relevant.

Another challenge is understanding factors that might characterize children from low SES families who, despite their adverse circumstances, are able to maintain healthy weight. Chen (2012) claims that research should focus on those who maintain good health and good health behaviors to identify protective factors (34). The evidence regarding protective factors against child obesity among children from low SES is extremely limited. One qualitative study among low SES parents of children from grades 5 to 7 revealed protective factors for child obesity, such as values and beliefs about gains from physical activity and healthy eating, enjoyment of activity and food, or child choice of foods (35). A systematic review has shown that higher parental resilience was a protective factor for child overweight and obesity in children from disadvantaged backgrounds (31).

The present study aimed to overcome gaps in the scientific literature by studying protective factors in addition to risk factors for child overweight and obesity in Israel. The population in Israel consists of two major population groups, Jews and Arabs (referring to Israeli Arab citizens living within Israel's boundaries before 1967). The Jewish majority are Israeli-born individuals, mostly descendants of immigrants, or immigrants from diverse countries. Arabs are native-born, who became a minority after the 1948 war and the establishment of the State of Israel, of whom 82% are Muslim, 9% are Christian, and the rest Druze or others (36). The two population groups differ in many socio-economic, social, and cultural ways. The average educational attainment and income level of the Arab population remain below that of the Jewish population, unemployment rates and participation in the work force are much lower, particularly among women, and poverty is very high (54% compared with 15% of Jewish families) (37). More than half of the Arab population resides in villages or small towns in the geographic periphery, while the majority of Jews live in urban areas (37). Fertility rates, although dropping in the last decade among Arab women, are still higher (3.4 births) than for Jewish women (2.9 births) (37). Socioeconomic and social inequalities between the two populations are reflected in health disparities. For example, poor self-reported health (38), mortality, and non-communicable diseases (39) are higher among Arabs compared to Jews, and obesity is particularly prevalent in Arab women (40). As in other countries, the Israeli population is facing a rising prevalence of child obesity. By 2015, about 25% of children in school grades 7–12 were overweight or obese, with rates higher in Arab than Jewish children (9).

To summarize, a multitude of studies indicating that risk factors are more prevalent among children in low socio-economic groups stand in sharp contrast to the limited scientific knowledge of factors that protect against obesity in low SES populations. In Israel, evidence on factors contributing to child obesity is scarce and mainly restricted to maternal BMI as a risk factor (40) and child physical activity as protective (41). There is no information regarding factors in low SES populations and how they differ between Jewish and Arab population groups. The current study aimed to extend our understanding of risk and protective factors for child overweight and obesity (hereafter overweight/obesity) taking a comprehensive biopsychosocial approach within each population group and focusing on children from low socio-economic backgrounds. The specific study objectives were to assess the prevalence of biological, behavioral and psychosocial factors for child overweight/obesity in Jewish and Arab population groups, and to compare their associations with child overweight/obesity in the two groups.

## Materials and methods

### Study design and population

This cross-sectional study was conducted among 5–6 years old children registered in Mother and Child Health (MCH) clinics serving low-socio-economic localities in the northern region of the Israel Ministry of Health. MCH clinics in Israel provide a universal free service and are used by the majority of the population. In the northern region, MCH services are provided by the Ministry of Health alone to the Arab population, while the services to the Jewish population are divided between the Ministry of Health and health maintenance organizations. The northern region lies in a peripheral region of Israel, with similar numbers of Jews and Arabs living in small towns or villages of low SES ranking. The study was approved by the Helsinki Supreme Committee of Israel Ministry of Health.

### Sampling and data collection procedures

In order to control for known disparities in economic circumstances between the Jewish and Arab populations in Israel (37) localities of low socio-economic clusters were selected for each population group (2–5 and 1–3, respectively, in the classification of local authorities) (42). A sample of MCH clinics was constructed from localities that represent the background data distribution (socio-economic cluster of the locality, size of locality, type of locality) in each population group as well as religion in the Arab population (Muslim, Christian, and Druze) and religiosity (secular/religious or ultra-orthodox) in the Jewish population. A total of 20 clinics were included, 8 in Jewish localities and 14 in Arab localities. There were some constraints that prevented the inclusion of some clinics, such as the lack of physical space for testing and interviews, and therefore the sampling was not entirely random. Within each sampled MCH clinic, a random sampling of children in the year prior to school entry (5–6 years old) and registered in the clinic was carried out, with each third child chosen to participate in the sample.

Data collection for the study was carried out as part of a “One Stop Shop,” a special project conducted by the Regional Health Bureau in the northern region, where preschool children were invited for assessment of their readiness for school in terms of hearing, vision, weight, height, development, and vaccinations. During spring and summer of 2015, the clinic nurses contacted the mothers by telephone and invited them to attend with their child. Of the 1112 children attending the sessions (representing 58 and 75% of the invited Jewish or Arab population respectively), 1001 (90%) agreed to participate in the study and after written informed consent was obtained, completed questionnaires relating to potential obesity risk and protective factors: 83.3% of Jewish mothers (399 out of 479 children), and 95.1% Arab mothers (602 out of 633 children). The main reason given for nonresponse was lack of time. At each MCH clinic, the nurses also filled out data on pregnancy, birth and infant nutrition retrieved from the children's clinic records. Questionnaires completed by fathers/grandmothers (38), two of children with growth hormone deficiency, and partially completed questionnaires (17) were excluded from data analysis. The final sample was 946 children (371 and 575 in the Jewish and Arab groups respectively).

### Measures

Variables were obtained from the mother, unless otherwise specified.

*Dependent variable*—child BMI: current weight and height were measured by the nurse and registered at the time of the visit. The children were weighed without shoes in light clothing, using electronic or non-electronic scales (depending on availability at the clinic). Height was measured in all clinics with a height gauge fixed to the wall, without shoes, feet together, knees straight, and head erect. Weight and height were transformed into BMI-for-age z-scores using WHO references 2006 for child's sex and age (43), then the corresponding z-scores were converted to percentile according to percentile- Z–score conversion values (10) and were grouped into a three-level variable: healthy weight (BMI% <85%), overweight (BMI% ≥85% <95%) and obesity (BMI% ≥95%). For the analysis it was further dichotomized into healthy weight “0” vs. overweight/obesity “1.”

Child and mother demographic background variables*:* Taken from the child's clinic record and completed as necessary from the mother, included child's date of birth and sex, maternal age, country of birth, year of immigration, marital status, number of children.

*Explanatory variables*: The variables were composed as variable groups, as follows:

Biological variables: The child's birth weight (taken from the record) and transformed into z-scores; maternal BMI was calculated as kg/m^2^ based on height and weight measured during the current visit by the nurse or self-reported.

Mother and family SES: Mother's education and family income.

Child nutrition, physical activity: infant feeding at 6-months (exclusive breastfeeding, supplemented, or formula fed) (taken from the record). Current nutrition was measured by several variables, adapted from a questionnaire from the HENRY program in England and originally derived from the Hammond food frequency questionnaire (44): (a) 8 types of food and frequency of eating portions per day or week—fruit; vegetables; bread, rice, potatoes, pasta; meat, fish, eggs, and beans; milk and dairy; high fat and sugar; soft drinks; water. Average frequency per week for each type of food was calculated. (b) Eating behaviors were assessed by the number of snacks the child habitually eats between meals, grouped into 4 unhealthy (e.g., chips, sweets) or 4 healthy snacks (e.g., fruit, vegetables, nuts). An additional four items asked about the child's eating habits, such as eating regular meals, mother allowing child to eat what and when s/he wants, on a Likert scale (never/hardly ever/sometimes/often/almost always). The items did not form a reliable scale (Cronbach's α = 0.43) and we used a single question (“You let the child eat whenever he wants during the day”). Physical activity of the child (frequency of physical activity and play) was assessed by a single question, with 5 response categories (none/< 1 h/ about 1 h/ 2 h/ 3+ h per day). Sedentary behavior of the child (number of hours of watching television / surfing / computer games), a single question with 6 response categories (none/< 1 h/ 1–2 h/ about 3 h/ 3–4 h/>4 h per day).

Child general behavior was assessed by the Strengths and Difficulties Questionnaire (SDQ) (45), divided into two sub-scales of difficulties (20 items) and pro-social behavior (5 items). Response categories for each items are “0” not true, “1” somewhat true' and “2” certainly true, and are summed to form the final score (0–30, and 2–10, respectively for each sub-scale in the current study). Cronbach's α values in the study were 0.80 and 0.53, respectively.

Maternal psychological attributes: Mother's general resilience was assessed by the Connor-Davidson Resilience Scale 10 (CD-RISC 10), a 10-item scale about the individual's general resilience in stress situations (46). Each item is scored from “0” not true, to “4” always, summed to form the final score. Cronbach's α value was 0.83 in the current study and the score range was 8–40. Maternal confidence as a parent was adapted from the HENRY questionnaire, originally derived from the Dumka parenting self-agency questionnaire (44), a scale of 4 items relating to the mother's confidence, knowledge and willingness to expend effort in problem-solving with their child e.g., “I feel sure of myself as a mother,” and were completed using a Likert scale, “1” never, to “5” always, with a mean score of the 4 items. Cronbach's α value was 0.74 in the current study. Maternal self-efficacy regarding the child's lifestyle was measured by 6 items asking the mothers to rate her ability to encourage the child's good behavior and setting limits in relation to mealtimes, snacks, TV and computer games, active play, bedtime (5 of the 6 items from the HENRY questionnaire (44), and an adapted item about her ability to generally explain which are healthy foods, Cronbach's α = 0.74. Maternal self-efficacy to maintain her own healthy lifestyle was measured by 3 items, regarding her ability to maintain balanced weight, to be routinely physically active and to resist food temptations, Cronbach's α = 0.73.

Maternal lifestyle: Physical activity was assessed by a single question about the frequency of various physical activities, of at least 30 min duration (no activity/<1 a month/1–2 times a month/ 1–2 times a week/ 3–4 times a week/>3 times a week). Sedentary behavior (screen time–watching TV, computer movies, games or surfing), a single question identical to that of the child (none/< 1 h/ 1–2 h/ about 3 h/ 3–4 h/>4 h per day). Maternal smoking during pregnancy (yes vs. no) was retrieved from the clinic records, and current smoking (yes vs. no) was reported by the mother.

Division into risk factors or protective factors was considered only for variables with some evidence in previous research. Risk factors included high BMI of the mother, high birth weight, child behavioral difficulties, child nutrition–fatty / sugary foods, soft drinks, unhealthy snacks, child and maternal sedentary activities, maternal smoking. Protective factors included breastfeeding, child physical activity, maternal confidence as a parent, maternal self-efficacy (for child's lifestyle), child nutrition–fruits and vegetables, healthy snacks. No clear direction was determined for other measures.

### Statistical analyses

All the analyses were performed using SPSS version 23. First, scales were constructed and initial analyses were conducted. Second, in an effort to reduce the number of variables, the 8 snacks were grouped into two variables of healthy and unhealthy snacks, and factor analysis of 7 types of foods was carried out (water was excluded as there was no variance, with almost all children drinking >1 cup/day). Principal Axis Factoring (PAF) technique was used to perform an exploratory factor analysis (EFA) yielding three factors that were similar in both groups: (1) carbohydrates/proteins/dairy–bread, rice, potatoes, pasta/ meat, fish, eggs, and beans/ milk and dairy; (2) fruit/vegetables; (3) high fat and sugar foods/soft drinks (See Supplementary Material and Supplementary Table 1) The score for each factor was computed as the mean frequency of eating these foods per week. Third, the differences in background demographic variables and in all the study variables by group were examined using the independent *t-*test or Chi-square test. Finally, multivariate analysis to assess variables associated with child overweight/obesity was carried out after an imputation process because the combination of the missing values of all the variables included in the analysis reduced the sample by about 20%. Description of the imputation process is available in Supplementary Material 2.

The multivariate analyses were performed separately in each population group using logistic regression models to estimate the likelihood of child overweight/obesity vs. healthy weight. In preparation for the analysis we examined the correlation matrix of all the study variables see (Tables 1, 2) in each group. Only the correlation between smoking during pregnancy and current smoking among Jewish mothers exceeded 0.5 (*r* = 0.559, *p* < 0.001) and we opted to use current smoking only in this group. Our approach for selecting which and at what stage variables were included in the regression models was based on our aim to examine the contribution of a combination of behavioral and psychosocial factors to child overweight/obesity in addition to the known socioeconomic and biological factors. Therefore, the logistic regression was conducted in stages: SES and biological variables together with universal demographic factors were included in the first stage (Model 1) and behavioral and psychological variables in the second stage (Model 2), using the Enter method. Interaction terms were included in the third stage using the Stepwise method to assess moderation effects, variables that alter the associations of risk factors with child obesity (Model 3).

**Table 1:** Demographic background variables in the Jewish and Arab population groups.

**Variable**	**Jews *n* = 371**	**Arabs *n* = 575**	***p*-values^a^**
**CHILD**			
Age (years)	6.03 (0.31)	5.89 (0.28)	<0.05
Gender (male)	181 (49%)	293 (51%)	ns
**MOTHER AND FAMILY**			
Age (years)	35.9 (5.1)	33.0 (1.3)	<0.001
Children in the family (*n*)	3.3 (1.7)	3.3 (1.3)	ns
Marital status (married)	336 (94%)	560 (99%)	<0.001
Education (years)	13.8 (2.1)	13.0 (2.7)	<0.001
Family monthly income (ILS)^b^			<0.001
<4,500	36 (10%)	106 (19%)	
4,501–7,500	72 (21%)	240 (43%)	
7,501–11,000	103 (29%)	110 (20%)	
11,001–16,000	86 (25%)	68 (13%)	
>16,000	53 (15%)	29 (5%)	

Data are presented as mean ± sd or percent;

a *p-values for t-test or Chi-square statistics*.

b *$1 = 3.5 ILS, Israeli Shekel*.

**Table 2:** Comparison of the study variables in the Jewish and Arab groups.

**Variable**	**Jews *n* = 371**	**Arabs *n* = 575**	***p*-values^a^**
**CHILD OVERWEIGHT/OBESITY (DEPENDENT VARIABLE)**			
BMI ≥85th centile	94 (25%)	109 (19%)	<0.02
**MATERNAL WEIGHT**			
Maternal BMI (kg/m^2^)	24.9 (4.8)	26.1 (5.0)	<0.001
**INFANT DATA**			
Birthweight (kg)	3.240 (0.533)	3.227 (0.510)	ns
Breastfeeding (at 6 months)	257 (71%)	489 (86%)	<0.001
**CHILD'S NUTRITION, ACTIVITY, BEHAVIOR**			
Food (frequency per week)			
Fruit /vegetables	25.4 (14.0)	33.4 (17.8)	<0.001
Bread, rice, potatoes, pasta/ Meat, fish, eggs, beans/ Milk, and dairy	34.6 (17.4)	36.0 (22.2)	ns
High fat and sugar/soft drinks	14.5 (12.6)	20.8 (15.8)	<0.001
Snacks^b^			
Healthy snacks	1.9 (1.1)	2.5 (1.2)	<0.001
Unhealthy snacks	2.1 (1.2)	2.6 (1.3)	<0.001
Child allowed to eat whenever wants to^c^	2.4 (1.0)	2.4 (0.9)	ns
Physical activity (≥2 h/day)	174 (48%)	398 (69%)	<0.001
Sedentary activity (≥3 h/day)	50 (14%)	111 (20%)	<0.05
Behavioral difficulties	7.4 (5.2)	9.8 (5.3)	<0.001
Prosocial behavior	8.5 (1.5)	8.4 (1.6)	ns
**MATERNAL PSYCHOLOGICAL ATTRIBUTES**			
Resilience	32.2 (5.7)	30.7 (6.1)	<0.001
Maternal confidence	4.6 (0.4)	4.4 (0.5)	<0.001
Self-efficacy regarding child's lifestyle	4.1 (0.6)	4.0 (0.6)	<0.01
Self-efficacy regarding own lifestyle	3.5 (0.8)	3.6 (0.8)	<0.05
**MOTHER'S LIFESTYLE**			
Physical activity (≥3–5 times/week)	89 (24%)	144 (26%)	ns
Sedentary activity (>3 h/day)	32 (9%)	54 (9%)	ns
Smoking in pregnancy	43 (12%)	8 (1%)	<0.001
Current smoking	80 (22%)	10 (2%)	<0.001

*Data are presented as mean ± SD or percent*.

a *p-values for t-test or Chi-square statistics*.

b *Number of each type of 4 snacks eaten between meals*.

c *Range “0” never, to “4” almost always*.

In the first stage, SES (maternal education, family income), and biological variables (maternal BMI, child birth weight z-scores) were included as relevant control variables together with universal demographics (child's age and gender and maternal age), a total of 7 variables. Number of children in the family, marital status, and infant breast feeding were not related to child overweight/obesity and were excluded from further analysis. In the second stage the explanatory behavioral and psychosocial variables were included. However, in order to reduce the number of variables, preliminary regression models were performed to evaluate the effect of factors from groups of child's or mother's variables on the odds of overweight/obesity. In each population group, adjusting for the seven control variables, the child's nutrition and behavior variables, and mother's two groups of variables (psychological attributes; lifestyle) were included in a second stage. Only variables significantly related to child overweight/obesity at *p* < 0.05 level in both or one of the population groups were included in the second stage of the final logistic regression (see Table 3) in order to have a common set of variables for both population groups. Variables were entered using the Enter method. In the third stage, interaction terms were examined and the significant interactions were then assessed separately using the PROCESS procedure, moderation model (Model 1) (47).

**Table 3:** Predictors of child overweight/obesity in Jewish (*n* = 364) and Arab (*n* = 572) children.

	**Jews**		**Arabs**	
	**Model 1**	**Model 2**	**Model 1**	**Model 2**
**Variable**	**OR (95% CI)**	**OR (95% CI)**	**OR (95% CI)**	**OR (95% CI)**
**SES AND BIOLOGICAL FACTORS**				
Maternal education	**0.88 (0.78, 1.00)**	0.94 (0.82, 1.08)	1.03 (0.94, 1.13)	1.03 (0.94, 1.14)
Family income^a^	**1.32 (1.06, 1.65)**	1.27 (1.00, 1.62)^#^	1.00 (0.81, 1.25)	0.99 (0.80, 1.25)
Maternal BMI (kg/m^2^)	**1.10 (1.04, 1.15)**	**1.10 (1.04, 1.16)**	**1.09 (1.04, 1.13)**	**1.08 (1.04, 1.13)**
Birth weight (z-score)	**1.31 (1.04, 1.66)**	**1.29 (1.01, 1.65)**	**1.35 (1.09, 1.66)**	**1.36 (1.09, 1.69)**
**CHILD'S NUTRITION, PHYSICAL ACTIVITY, BEHAVIOR**				
Food (frequency per week):				
Fruit /vegetables		1.00 (0.98, 1.02)		1.00 (0.98, 1.01)
Bread, rice, potatoes, pasta/ Meat, fish, eggs, beans/ Milk, and dairy		0.99 (0.97, 1.00)		1.00 (0.99, 1.02)
High fat and sugar/soft drinks		1.01 (0.99, 1.04)		1.00 (0.99, 1.02)
Snacks–unhealthy		**0.76 (0.60, 0.96)**		**0.81 (0.68, 0.97)**
Child allowed to eat whenever wants to^b^		0.85 (0.64, 1.11)		**0.69 (0.53, 0.89)**
Physical activity (≥2h/day)^c^		0.59 (0.34, 1.03)^*##*^		0.83 (0.52, 1.33)
Child's behavior difficulties		**1.09 (1.02, 1.15)**		0.97 (0.93, 1.02)
**MATERNAL PSYCHOLOGICAL ATTRIBUTES**				
Maternal resilience		**1.07 (1.01, 1.13)**		1.02 (0.98, 1.06)
Maternal self-efficacy regarding child's lifestyle		**0.46 (0.27, 0.80)**		**0.48 (0.31, 0.75)**
**MATERNAL LIFESTYLE**				
Current smoking (yes)		1.44 (0.78, 2.68)		Not included
Chi^2^ (df)	32.22 _(7)_^***^	70.33 _(17)_^***^	28.75 _(7)_^***^	51.25 _(16)_^***^
Nagelkerke R^2^	0.124	0.258	0.079	0.138

*Overweight/obesity defined as 1 = overweight/obesity, 0 = healthy weight; All models are also controlled for child's age and gender, and maternal age. A high score on each attribute indicates a higher level, unless otherwise defined; OR, odds ratio; CI, confidence interval; df, Degreed of Freedom*.

*OR values significant at p ≤ 0.05, p ≤ 0.01 or p ≤ 0.001 are in **bold font***.

a *Included on a 5-level range, “1” lowest, to “5”highest*.

b *Included on a 5-level range, “0” never, to “4” almost always*.

c *Reference: “0” 1 h or less/day*.

# *p = 0.057*,

## *p = 0.063*,

*** * p ≤ 0.001*.

## Results

### Description of study population and the study variables, by population group

The mean age (±sd) was higher for the Jewish children (6.03 ± 0.31) than the Arab children (5.89 ± 0.28, *p* < 0.05). There was an equal distribution of boys and girls in both groups. Most mothers were in their mid-thirties, with Jewish mothers significantly older than Arab mothers. Although almost all mothers were married, the rate of unmarried mothers was higher in the Jewish group. There were no differences between the groups in the number of children in the family. SES variables were significantly higher in the Jewish group; the mothers had higher mean years of education (only 3.3% had <12 years of education vs. 15% among the Arab mothers) and higher family income (Table 1). Of the Jewish mothers, 82% were Israeli-born, 18% immigrants (14% from the Russian Federation, most of them immigrated as children, and the rest from other countries); of the Arab mothers, 82% were Muslim, 5% Christian, and 13% Druze / Circassian (not listed in the table).

Description of the study variables for both groups is presented in Table 2. The total rate of child overweight/obesity (the dependent variable) was 22.5%. It was significantly higher among children in the Jewish group (25%), of whom 12% were with overweight and 13% with obesity, as compared with Arab children (19%), of whom 10% were with overweight, and 9% with obesity. There was no difference in birthweight between the groups, but duration of breastfeeding was significantly longer in the Arab children. Arab children compared to Jews consumed significantly more of the following foods—fruits, vegetables, fatty / sugary foods, and sugary drinks but did not differ from Jewish children in terms of frequency of eating carbohydrates, proteins and dairy foods. The Arab children ate more snacks—both healthy and unhealthy, and were more physically active but also more sedentary. They presented with more behavioral difficulties although there were no differences in pro-social behavior. Regarding maternal characteristics, variables were significantly worse among the Arab mothers: mean BMI was higher, reflected in significantly higher (20%) obesity rates (BMI ≥30 kg/m^2^) than among Jewish mothers (14%); the overall levels of resilience, maternal confidence and self-efficacy regarding their child's lifestyle were lower. They reported more physical activity but did not differ in sedentary behavior from the Jewish mothers, and very few Arab mothers smoked.

### Multivariate analyses

Preliminary logistic regression models were conducted separately in each group in order to examine the variables significantly associated with child overweight/obesity (see statistical analysis). Controlling for the seven variables (child's age and sex, maternal age, maternal education, family income, maternal BMI, and child's birth weight z-score) four sets of variables were separately included in the second stage: the child's breast feeding in infancy, current nutrition, physical, and sedentary behavior (9 variables), child's general behavior (2 variables), maternal psychological attributes (4 variables) and maternal lifestyle (3 variables) were included in a second stage (data not shown in tables).Variables significantly associated with child overweight/obesity in both or either the Jewish or Arab group in each analysis were included in the final models to form a common set of variables in the two groups. In both groups no significant associations with child overweight/obesity were found for breastfeeding, child prosocial behavior, eating healthy snacks, and sedentary activity, maternal confidence, maternal self-efficacy toward own lifestyle, and maternal physical and sedentary activity; they were excluded from the final analyses. It should be noted that maternal smoking was not examined in the Arab group due to the negligible number of smokers. Additionally, although only one type of food (carbohydrate/protein/dairy foods) was associated with child overweight/obesity in one population group, we decided to include all types of food in the final analyses in order to characterize an overall diet. Therefore, 9 variables were included in the second stage of the final regression model in both groups, with additional maternal smoking among Jewish children (Table 3).

A combination of risk factors and protective factors were found to explain overweight/obesity among the Jewish children (Table 3). In the first step, in addition to the statistically significant biological risk factors (higher maternal BMI and higher birthweight), maternal education was inversely associated and income was positively associated with child overweight/obesity. However, only the biological factors remained significantly associated with overweight/obesity, and income was of borderline significance (*p* = 0.057) once all other child and maternal variables were included in the second step. The likelihood of child overweight/obesity vs. healthy weight was lower when the child ate more unhealthy snacks, but none of the types of food the child ate were associated with overweight/obesity. The lower likelihood of child overweight/obesity when the child was more physically active, suggesting protective effects, was only of borderline significance (*p* = 0.06). Additionally, the child behavioral difficulties increased the likelihood of overweight/obesity. In terms of psychosocial and lifestyle behavior characteristics of the mother, we found that, contrary to expected, the likelihood of overweight/obesity was higher with higher general resilience, but, as expected, it was lower when the mother's self-efficacy regarding the child's healthy lifestyle was higher, suggesting a protective association. Maternal smoking did not significantly affect the likelihood of overweight/obesity. In both steps the regression models were significant but the additional contribution to the variance (represented by the Nagelkerke R^2^) in the second step was larger than in the first one.

**Figure 1:**
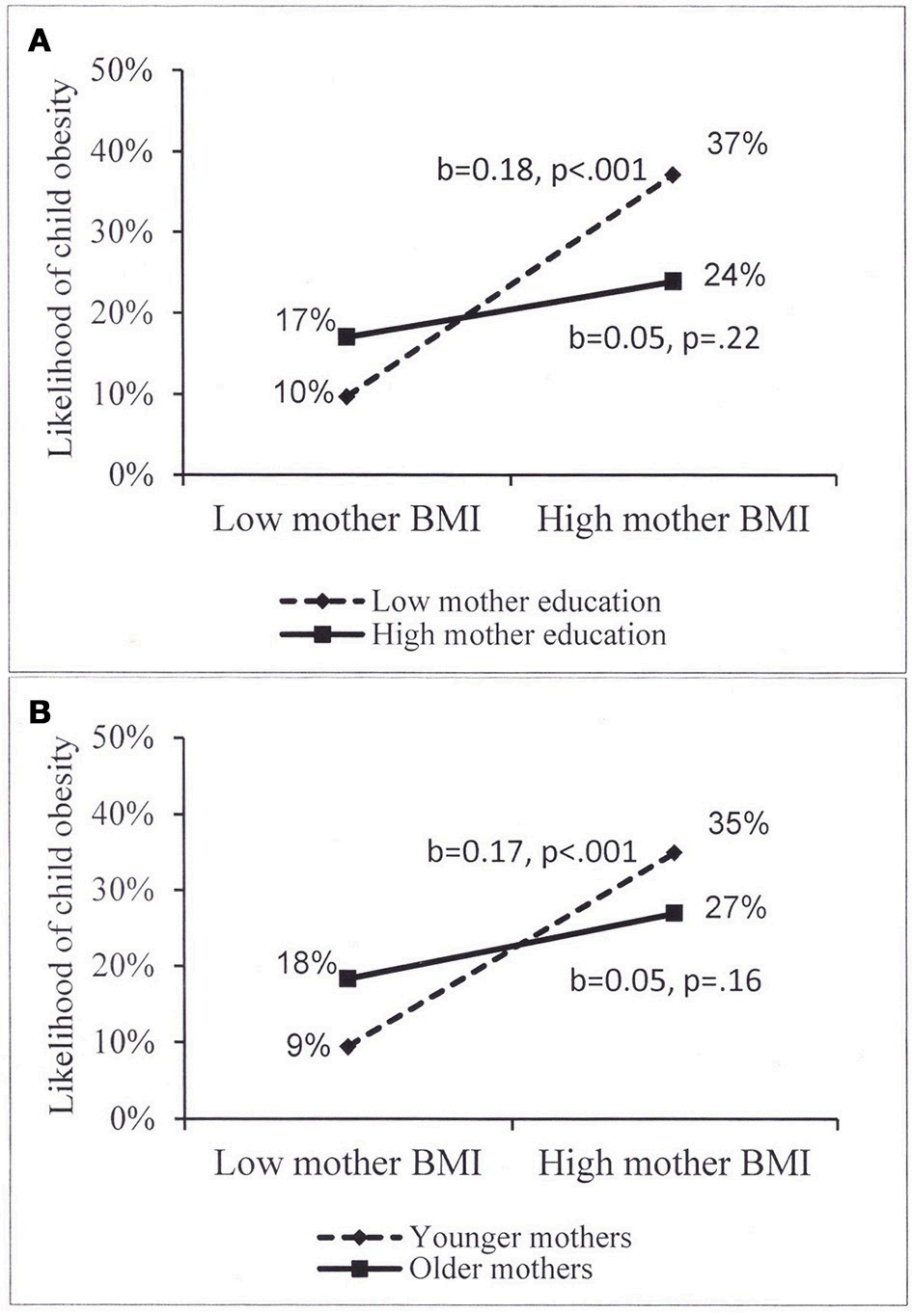
**(A,B)** Moderation of the association of mother BMI with child overweight/obesity among Jewish children, by **(A)** mother eduction, high eduction = more than 15 years of eduction; **(B)** mother age, older age = above 39.9 years of age.

In the final step, examining potential interactions, two significant interactions emerged. The association of maternal obesity with child overweight/obesity was moderated by maternal education (*p* = 0.014) and maternal age (*p* = 0.043). Examination of each interaction by the PROCESS procedure revealed that when maternal education was low, the positive association was strong and highly significant (*b* = 0.18, *p* < 0.001) and when maternal education was higher (more than 15 years of education), the association became nonsignificant (*b* = 0.05, *p* = 0.220) (Figure 1A). The examination of the second interaction (Figure 1B) showed that when mothers were young the positive association was strong and significant (*b* = 0.17, *p* < 0.001) but among older mothers (above 39.9 years of age), the association became nonsignificant (*b* = 0.05, *p* = 0.157).

Among the Arab children, fewer variables than those found in the Jewish children were significantly associated with overweight/obesity (Table 3). Maternal BMI and the child's birthweight were significant risk factors for overweight/obesity, similar to the findings in the Jewish children, while socio-economic factors were nonsignificant even in the first step. Of the other explanatory child factors, and contrary to expectations, eating unhealthy snacks and being allowed to eat whenever the child wanted lowered the likelihood of overweight/obesity. The frequency with which the child ate all types of foods were unrelated to overweight/obesity. As in Jewish children, maternal self-efficacy regarding the child's healthy lifestyle reduced the likelihood of overweight/obesity, the only maternal psychosocial significant explanatory and protective factor. No interaction terms were statistically significant. The regression models in both steps were significant but the contribution to the variance (Nagelkerke *R*^2^) was low.

## Discussion

The present study examined the prevalence of obesity and contribution of factors related to overweight/obesity among 5–6 years-old children from low socio-economic families in Israel, and compared these associations in the Jewish and Arab groups. The findings indicate that the prevalence of risk and protective factors differed between the two groups, and that the associations between the factors and overweight/obesity were only partially similar in the two groups.

In our sample of children from low socio-economic backgrounds, we found that more than 20% children were above the 85th centile for BMI. The rates are somewhat higher than the 18% national data from 2016 for 1st grade (6–7 years old) children (48). This is not surprising given the low SES level in our sample compared to national figures: close to 67% of the mothers in our sample attained up to secondary or less than higher education degree compared to 51% in 25–64 years-old (49). The average household income level of our participants was about NIS7500 (the upper bound of category 2), considerably lower than the national average household income of NIS18,671 and similar to the average income for decile 3 in the country (50). Furthermore, overweight/obesity rates in Jewish children were significantly higher than in Arab children, which also differed from the national data where rates are similar (48). This finding is comparable to the UK where higher rates are found in White British than Pakistani 3–years old minority children (17) but contrasts with US studies which show that obesity rates among 5–6 years old are higher in racial/ethnic minorities (4) or show no ethnic differences for children of low socio-economic status (33).

Our study sheds light on the prevalence of risk and protective factors in a way previously unexamined in preschool children in Israel. We found a complex pattern. Among Arab children, the levels of some risk factors were higher, namely in terms of general behavior difficulties, hours of sedentary behavior, and consumption of food – both unhealthy and healthy foods and snacks. They also had higher levels of protective factors such as prevalence and duration of breastfeeding and physical activity, reflecting the physical environment in Arab communities that allows for recreation outside. This is no doubt related to the poorer public transportation and infrastructure in Arab villages (37) where young children may have no alternative but to play in the streets and walk to pre-school and friends. The question of whether these factors contributed to the lower rate of overweight/obesity at this age requires further examination. Alongside these factors were lower levels of maternal protective factors, namely resilience, parental confidence, and especially self-efficacy with regard to child health behaviors. These differences may well reflect differences between the population groups in dietary patterns (51) and in behaviors and perception of control or resilience (52). It may be that a combination of these factors accumulate over the years and contribute to the higher overweight/obesity rates in Arab children, that occur by the age of 11–12 years (9). Longitudinal studies of preschool children examining not only the increasing obesity rates but also changes in risk and protective factors may provide answers to this issue. A potential additional factor might relate to the Jewish mothers' backgrounds which were likely to have been diverse. This could not be examined in our young study population (mean age about 35 years) as the vast majority were Israeli-born and data on their parents' country of origin was unavailable. This information is important in order to understand better the differences between the two population groups.

The multivariate analyzes revealed that our combination of biopsychosocial and behavioral factors for overweight/obesity was only partially similar in the two groups. Regarding SES, the variables carried a different risk for Arab and Jewish children, as maternal education and family income were significantly associated with overweight/obesity in the Jewish but not the Arab children, and only in the first stage of the analysis (Model 1). The attenuation of the associations to non-significance (maternal education) or to borderline significance (income) in Model 2, with the inclusion of psychosocial and behavioral factors, supports evidence that SES inequalities in health are explained by differences in the psychosocial environment and behaviors (53). Additionally, the initial protective effect of maternal education is similar to earlier evidence (21). However, our finding that higher income is a risk factor, even if only of borderline statistical significance, contradicts prior studies (7, 21). It is possible that this finding is specific to our population; namely that within a population of low SES, children from more affluent families are at higher risk. Some parallels are evident from a study of primary school children showing that the SES-child obesity association differs across five European countries. The association of low SES with overweight/obesity was found in the most affluent country (Sweden), but in the less affluent countries lower SES was associated with *lower risk* overweight/obesity (54). The lack of an SES-child overweight/obesity association in our Arab population group reinforces the need for further exploration.

In our study the known biological risk factors (maternal BMI and birth weight) were significantly associated with overweight/obesity. However, alongside the strong association of these two variables with overweight/obesity, mothers' self-efficacy in maintaining their children's healthy behaviors proved to be a distinct protective factor in both groups, suggesting that this variable may be a crucial focus for intervention across cultures. This finding supports previous evidence regarding maternal self-efficacy in 3-years olds in the UK (24) but contradicts others who found a nonsignificant association with BMI among 4-years olds in Sweden (55). Inconsistencies across studies may arise from different study designs and measures as well as from cultural differences. Other studies in which maternal self-efficacy was examined in relation to children's food intake and sedentary behavior (55, 56) seem to indicate that the relationship between maternal self-efficacy and child's overweight/obesity is more complex, and potentially mediated by the child's lifestyle behaviors. Although further studies are needed, the current findings suggest that interventions which target maternal self-efficacy early in their children's lives might contribute to childhood obesity prevention in both population groups in Israel. Focusing on the preschool years may be especially important as others have shown that maternal self-efficacy can decline over the first years of a child's life (29).

Our analyses revealed additional child and maternal risk and protective factors in the Jewish children that contribute to explaining overweight/obesity beyond SES and biological factors, while few variables were significant in Arab children. In the Jewish group, the inverse association of child physical activity with overweight/obesity was only of borderline significance, marginally supporting previous evidence of its protective role in a systematic review of studies among children from disadvantaged backgrounds (31). On the other hand, the assumption that mothers' resilience, a general personality characteristic that is not focused on eating or physical activity, is a protective factor was not supported, and was found to be associated with a higher likelihood of child overweight/obesity. In the absence of studies that include this indicator, it is possible that this personality characteristic may be protective only for mothers' own food intake (57) and weight. The higher likelihood of children with behavioral difficulties to be with overweight or obesity may reflect a bi-directional association and should be further explored; others have suggested that there is a threshold effect between behavioral difficulties measured by SDQ (which was used in our study) and BMI z-score (58).

Contrary to the expectation that eating fruit/vegetables is protective and consumption of fatty/sugary foods and soft drinks are risk factors in children we did not find associations in either group, corroborating the inconsistency reported in studies among children from disadvantaged backgrounds (31). Of course the children in our study are young and follow up over time is required to determine if preschool nutrition is a risk factor for later development of obesity. The additional inverse association of the child consumption of unhealthy snacks with overweight/obesity in both groups as well as the inverse association with the mother's feeding behavior (“letting the child eat whenever he wants”) in the Arab group suggest the possibility of a bi-directional association: mothers of children with overweight/obesity may be restricting access to unhealthy snacks and children's freedom to eat in an attempt to manage their weight. Maternal smoking, representing exposure to secondhand smoke, has been suggested as a risk factor for child overweight/obesity (21) but our findings in the Jewish group did not support this. We were unable to determine the effect of smoking in pregnancy given the very low levels in both groups.

In both groups our assumption that the effects of biological risk factors on child overweight/obesity would be moderated by protective factors was generally not supported. The exception was for maternal education in the Jewish group. In this group, maternal education did not directly contribute to child overweight/obesity, but only indirectly by moderating the risk of high maternal BMI, and it had no contribution to overweight/obesity in the Arab children. This is an important finding suggesting that children of mothers with obesity and with lower education levels are at especially high risk for overweight/obesity, and that intervention programs need to be targeted toward less educated mothers and adapted to these differences. The additional moderation by maternal age showing that the association is strong and significant only in younger mothers is an interesting finding and is difficult to compare as most studies use age only as a control variable. It may be that this finding, unique to Jewish mothers of low socioeconomic background, reflects the probability that these younger mothers, despite having somewhat higher education level than older mothers (data not shown), are likely to have been brought up on a less healthy diet and more sedentary lifestyle, consistent with changes that have occurred in the last decade.

Among the protective factors that were not associated with healthy weight, our finding for breastfeeding as nonsignificant is perhaps not surprising given the evidence from two comprehensive reviews that the relationship is inconsistent (19, 31). This may be related to the high proportion of mothers still breastfeeding at 6 months, although it has been suggested that this is ethnically dependent and may be protective only in white European populations (31). It is harder to arrive at conclusions as to why maternal confidence was not found to be significantly protective even in the preliminary analyses, but it may be partially due to some overlap of the concept and measure of maternal self-efficacy.

### Limitations and strengths

Several limitations of the study should be noted. Its cross-sectional design makes it impossible to draw conclusions about causality (except for the effect of birth weight and breastfeeding), and associations may be bi-directional, particularly between maternal feeding practices (allowing the child to eat whenever s/he wants to) or food (unhealthy snacks) intake and overweight/obesity. In addition, since the study was conducted as part of a clinic visit, the questionnaires had to be brief, thus posing issues of validity as some of the instruments did not produce reliable scales and single items were used. Other issues common in any self-report study include reporting bias and social desirability. It is not possible to assess the direction of these biases between Jewish and Arab mothers. Strengths of the study include the random selection made for children within each MCH clinic and the careful selection of MCH clinics to represent the communities of lowest SES in diverse localities in the Northern region of Israel. The response rate of participants was gratifyingly high especially that of the Arab mothers. In our analysis we used imputation techniques to improve the integrity of the data.

### Conclusion and implications

Our findings provide a contribution to scientific knowledge of protective factors for healthy weight alongside risk factors for obesity among children from populations of low socio-economic status. We found that alongside some significant similar factors, the two population groups differed. Fewer factors were found in the Arab group suggesting that perhaps some measures were less suitable for them. Cultural and other factors affecting diet, activity, and weight, not examined in the current study, should be explored.

This study is the first in Israel to explore a comprehensive set of both protective and risk modifiable factors for overweight/obesity in preschool children in each population group. The evidence that obesity incidence between age 5 and adolescence is more likely to occur at younger ages (4) and the recommendation that child obesity should be monitored in low income preschool children due to their greater risk (58, 59), emphasize the importance of using our data as a basis for future follow-up studies in the North of Israel and other regions in the country. The findings may be relevant for understanding factors contributing to overweight/obesity in low SES populations of different ethnic backgrounds in other countries too.

Our findings provide a basis for recommendations regarding intervention programs in low-income populations and calls for strengthening protective factors rather than focusing on reducing risk factors alone. While it was clear that maternal BMI and birthweight were the most powerful factors associated with obesity, they are notoriously less amenable to change. Our finding, that maternal self-efficacy was a strong protective factor across both groups is important especially as it is potentially modifiable. We believe it should be a major component of interventions alongside other factors in both groups. Identification of the differences in the relationship between protective and risk factors in both Jewish and Arab children and mothers may lead to the design of more culturally appropriate intervention programs for each population group. They should start in the preschool years or even earlier, as the evidence is clear that “early interventions targeted toward disadvantaged children have much higher returns than later interventions” [(60), p.1902]. These recommendations may help to reduce the inequality of child obesity in the Northern Region in Israel, with implications for disadvantaged populations elsewhere.

## Ethics statement

This study was carried out in accordance with the recommendations of Israel Ministry of Health, Helsinki Supreme Committee of Israel Ministry of Health. The protocol was approved by the Helsinki Supreme Committee of Israel Ministry of Health. All subjects gave written informed consent in accordance with the Declaration of Helsinki.

## Author contributions

VS and MR had the initial idea for the study and wrote the initial draft of the paper. VS, MR, and MC-D outlined the first design of the study. All authors participated in the final planning of the study. NC monitored the data collection. VS and MR analyzed the data. All authors contributed to conceptualizing, designing and interpreting the analyses, and redrafting the paper. All authors approved the final version.

### Conflict of interest statement

The authors declare that the research was conducted in the absence of any commercial or financial relationships that could be construed as a potential conflict of interest. The reviewer SM and handling Editor declared their shared affiliation.
