# Supplementary material for: Risk and Protective Factors for Child Overweight/Obesity Among Low Socio-Economic Populations in Israel: A Cross Sectional Study
Source: Front Endocrinol (Lausanne). 2018 Aug 21;9:456. doi: 10.3389/fendo.2018.00456 (PMC6113577; doi:10.3389/fendo.2018.00456)
Supplement: Supplementary file 1 [file Data_Sheet_1.docx]

**Supplementary material 1: Factor analysis**

The Principal Axis Factoring (PAF) technique was used to perform an exploratory factor analysis (EFA), with Promax rotation, where the rotation assumed correlations to some extent between extracted factors (s1). Based on the eigenvalue criterion (ev>1), three factors were defined, which explained 51.7 and 52.8 of the variance among Jewish and Arab groups, respectively. Items in each final factor showed loading values above .35 (see the pattern matrix Supplementary Table 1). As the PAF technique allows for correlations, these were .54, .54, and .40 among all pairs of factors for the Jewish participants, and .51, .60, and .37 for the Arab participants.

Supplementary Table 1: Factor loading matrix of child‘s type of food, in Jewish and Arab sectors

|  | **Jewish** | | | **Arab** | | |
| --- | --- | --- | --- | --- | --- | --- |
|  | Factor 1 | Factor 2 | Factor 3 | Factor 1 | Factor 2 | Factor 3 |
| Proteins - Meat, fish, eggs, beans | **.805** | -.077 | -.114 | **.808** | -.040 | .009 |
| Carbohydrates -Bread, rice, potatoes, pasta | **.661** | -.015 | .159 | **.494** | .030 | .231 |
| Milk and dairy | **.568** | .177 | .043 | **.602** | .111 | -.086 |
| Vegetables | -.016 | **.881** | -.086 | .030 | **.855** | -.037 |
| Fruit | -.005 | **.604** | .100 | .020 | **.627** | .059 |
| High in fat /in sugar | -.026 | .010 | **.878** | -.109 | .064 | **.860** |
| Soft drinks | .003 | -.021 | **.497** | .230 | -.089 | **.485** |

**References**

s1. Hefetz A. Liberman G. The Factor analysis procedure for exploration: a short guide with examples.  *Culture Educ*, 2017;29 (3):526-62. Doi: 10.1080/11356405.2017.1365425

**Supplementary material 2: Imputation process**

Before the final stage of multivariate analyses we carried out a complex procedure to test and impute missing values, separately in each population group. The rates of missing values were somewhat lower in the Arab than in the Jewish group, but they were low across most study variables (smaller than five percent), except for maternal BMI among Jewish mothers (12.5 percent). This required one round of imputation (s2). The analyses for missing value pattern showed that missing was completely at random for the Jewish group (MCAR, Little's test: χ^2^=8,435.13, df=8,232, p=.058), whereas missing pattern for the Arab group did not show a random pattern (MNAR, Little's test: χ^2^=12,756.53, df=12,144, p<.001). Thus, the next step was to identify differences in missing value patterns in the Arab group, across groups of maternal education. A division between education categories (less than 12 years, high school education, and higher education) yielded a random pattern for each category (Lowest: MCAR, χ^2^=3,280.14, df=3,248, p=.342; Middle: χ^2^=7,866.57, df=7,833, p=.392; Highest: χ^2^=4,167.73, df=4,131, p=.341). Our imputation for the Arab group was conducted separately for each of these education categories (s3). Using the fully imputed data, a multivariate analysis to assess variables associated with child overweight/obesity was performed.

**References**

s2. Little, T. D. Design Issues in Longitudinal Studies. In: T. D. Little (ed.) *Longitudinal Structural Equation Modeling*. New York: The Guilford Press, 2013. pp. 37 - 70.

s3. Bar, H. Missing data – mechanism and possible solutions. *Cult Edu*, 2017, 29 (3):, 492 – 525. doi: 10.1080/11356405.2017.1365426

**Supplementary material 3: Data**
